# MiR-133b regulates bladder cancer cell proliferation and apoptosis by targeting Bcl-w and Akt1

**DOI:** 10.1186/s12935-014-0070-3

**Published:** 2014-07-19

**Authors:** Xiao-nan Chen, Ke-feng Wang, Zhen-qun Xu, Shi-jie Li, Qiang Liu, Dong-hui Fu, Xia Wang, Bin Wu

**Affiliations:** 1 Department of Urology, Shengjing Hospital of China Medical University, Shenyang 11004, Liaoning, P.R. China

**Keywords:** miR-133b, Bcl-w, Akt1, Bladder cancer, Proliferation, Apoptosis

## Abstract

**Background:**

MiR-133b is a muscle-specific microRNA; it has a role in the formation of cardiocytes and the expression of myocardium ion channels by regulating target genes. Many human malignant tumors demonstrate a low expression of miR-133b, as noted in colorectal, lung, esophagus and bladder cancers, but the role of miR-133b in bladder cancer is unknown.

**Methods:**

The expression of miR-133b in clinical bladder cancer specimens and adjacent normal tissues was confirmed by stem-loop RT-PCR. We also analyzed the relationship between miR-133b expression and clinicopathological factors of bladder cancer. Bcl-w and Akt1 protein expression in 41 bladder cancer specimens and adjacent normal tissues was detected by Western blot. After transfection of miR-133b mimics or inhibitor into a T24 human bladder cancer cell line, Bcl-w and Akt1 protein and mRNA expression were examined by Western blot and RT-PCR, respectively. The effect of miR-133b on T24 cell proliferation and apoptosis was measured by CCK-8 tests and flow cytometry, respectively.

**Results:**

The expression of miR-133b in bladder cancer tissues from 41 patients was significantly down-regulated (*P* < 0.01); low expression of miR-133b was strongly associated with high-grade bladder cancer (*P* < 0.01). Bcl-w and Akt1 proteins were significantly overexpressed in bladder cancer tissues versus adjacent normal tissues (*P* < 0.01 for both). The expression of Akt1 and Bcl-w proteins and Akt1 mRNA, in T24 cells was significantly down-regulated or up-regulated after transfection of miR-133b mimics or inhibitor, respectively; however, there was no significant difference in Bcl-w mRNA expression. Transfection of HEK-293 T cells with miR-133b significantly suppressed a luciferase-reporter containing the Bcl-w or Akt 1 3′-untranslated regions. MiR-133b mimics significantly inhibited T24 cell proliferation, as well as increased T24 cell apoptosis (*P* < 0.05 and *P* < 0.01, respectively) while the miR-133b inhibitor increased and decreased these, respectively (*P* < 0.05 for both).

**Conclusions:**

MiR-133b may play a very important role in the proliferation and apoptosis of T24 cells by regulating the expression of Bcl-w and Akt1.

## Introduction

Bladder cancer is the most common malignant tumor of the urinary system among Chinese. Most tumors (95%) originate from the urothelium, but the pathogenesis of bladder cancer has been unclear to date. While morbidity due to bladder cancer has been increasing on a yearly basis, the main treatment strategy has been surgery. However, the recurrence rate of bladder cancer is high, and advanced cases are usually complicated by local invasion and distal metastases, with poor outcomes. Understanding the molecular mechanisms of bladder cancer may contribute to innovative treatments.

MicroRNAs (miRNAs) are short (20–24-nt) single chain, non-coding RNAs that exist in eucaryons; their expression differs according to the specific tissue and developmental stage of individuals. They also have important regulatory functions during development and differentiation. By targeting 3′ untranslated regions (3′UTRs) of cognate mRNAs,miRNAs are involved in the translation or direct degradation of mRNAs, and thus regulate gene expression [1],[2]. Some researchers have shown that more than 50% of miRNA genes are located in regions related to tumors. In addition, different kinds of tumors express specific miRNAs, which may indicate that miRNAs have oncogene- or anti-oncogene-like functions and may play a role in the development of human tumors [3]. MiR-133b is a type of muscle-specific microRNA; it takes part in the formation of cardiocytes and the expression of myocardium ion channels by regulating target genes. Many human malignant tumors, such as colorectal,lung, esophagus and bladder cancer [4]-[7], express low levels of miR-133b; however, the role of miR-133b in bladder cancer is still unclear.

Bcl-w (also named BCL2L2) is a member of the Bcl-2 protein family and has a molecular weight of 22 kDa. The Bcl-2 gene family is made up of many members, including apoptosis inhibiting (e.g. Bcl-2, Bcl-xL, Bcl-w) and apoptosis promoting proteins (e.g. Bax, Bad) [8], which regulate programmed cell death. The proteins of this family form dimers that are involved in DNA damage or apoptosis in abnormal cells, such as tumor cells, by breaking the balance between apoptosis promoting and apoptosis inhibiting proteins [9]. Bcl-w shows higher expression in some epithelium-derived tumors, such as colorectal, cervical and breast [10]-[13]. However, reports in the literature relating to the expression and function of Bcl-w in bladder cancer are rare.

Akt1, a serine/threonine-protein kinase, also known as Akt kinase, is involved in the regulation of various downstream signaling pathways, such as those of cell metabolism, proliferation, survival, growth, and angiogenesis [14],[15]. A past study indicated that the Akt1 kinase was most frequently activated during proliferation and survival pathways in cancer [16]. Kim et al. [17] reported that [6]-shogaol reduced the constitutive phosphorylation of signal transducer and activator of transcription 3 (STAT3) and decreased the expression of cyclin D1/3, which are target proteins in the Akt signaling pathway in non-small cell lung cancer. Gai et al. [18] reported that ursolic acid is important in inducing apoptosis, via the suppression of Akt/NF-κB signaling in T24 human bladder cancer cells, and this occurs in a dose-dependent manner. Juanpere et al. [19] found that mutations in FGFR3 and PIK3CA, singly or combined with RAS and Akt1, were associated with Akt but not with MAPK pathway activation in urothelial bladder cancer. Previous reports have shown that Akt1 is involved in the regulation of bladder cancer; however, it is unclear whether miR-133b can regulate the expression of Akt1 in T24 human bladder cancer cells.

According to past bioinformatics prediction results, we speculated that Bcl-w and Akt1 might be target genes for miR-133b. In this study, we detected the expression of miR-133b, Bcl-w and Akt1 in clinical bladder cancer tissues. The functional role of miR-133b in bladder cancer and its influence on Bcl-w and Akt1 protein and mRNA expression, and cell proliferation and apoptosis, was studied after transfection of miR-133b mimics or inhibitor into a transitional human bladder carcinoma cell line, T24.

## Results

### MiR-133b expression is downregulated in bladder cancer tissues

**Figure 1:**
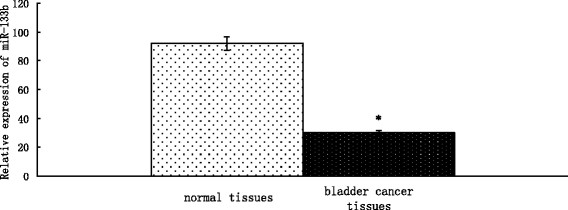
**MiR-133b expression is downregulated in bladder cancer tissue.** Expression levels of miR-133b were measured by stem-loop RT-PCR in bladder cancer specimens and corresponding, adjacent normal tissues. U6 RNA was used as an endogenous normalizer and the relative combined expression levels of miR-133b are shown. 2^-ΔΔCt^ values ± SEM, n = 41, *P < 0.01.

Expression levels of miR-133b were measured by stem-loop RT-PCR in 41 bladder cancer specimens and their corresponding adjacent normal tissues. Quantitative analysis indicated that miR-133b expression was significantly decreased in bladder cancer specimens compared to adjacent normal tissues (*P* < 0.01; Figure 1). These results implied that down-regulation of miR-133b may be involved in human bladder cancer disease processes.

### A negative relationship between miR-133b expression and the pathological grading of bladder cancer

Bladder cancer specimens were divided into low expression (below average expression of 30.4) or high expression groups (expression levels higher than the average of 30.4) according to the relative expression of miR-133b, normalized to U6 RNA. We found low miR-133b expression and high pathological grading were significantly negatively correlated (*P* = 0.003; Table 1). However, there was no significant association between miR-133b expression and gender, clinical stage or neoplasm recurrence (Table 1). These results suggested miR-133b down-regulation in human bladder cancer is strongly linked to a high-grade pathology.

**Table 1:** The relationship between miR-133b expression and clinicopathological characterisics of bladder cancer

**Charactistics**	**n = 41**	**MiR-133b level**		***P*** **value**
		**High (%)**	**Low (%)**	
**Gender**				
Male	29	11(64.5)	18(75)	0.475^a^
Female	12	6(35.3)	6(25)	0.187^a^
**Clinical stage**				
Ta-T1 (non-invasive)	24	12	012	
T2-T4 (invasive)	17	5	12	0.003^b*^
**Pathological grading**				
Low level	19	13	6	
High level	21	4	17	
**Neoplasm recurrence**				
Early-onset	25	10	15	0.812^a^
Recurrent	16	7	9	

^a^Chi-square test, ^b^Sided Fisher’s exact test, *P < 0.01.

### Determination of miR-133b sense and antisense transfection efficiency

**Figure 2:**
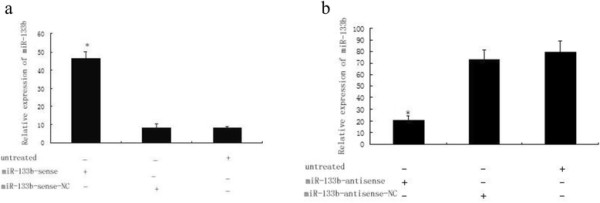
**Transfection of T24 cells with miR-133b sense and antisense.** The relative expression of miR-133b normalized to U6 RNA was determined by qRT-PCR. **(a)** MiR-133b expression was significantly increased after miR-133b mimics’ transfection into T24 cells. **(b)** MiR-133b expression was significantly decreased after miR-133b inhibitor transfection into T24 cells. *P < 0.01.

The relative expression of miR-133b in cells transfected by miR-133b mimics (sense) and inhibitor (antisense) was determined by qRT-PCR. We found the expression of miR-133b were significantly increased and decreased after transfection of miR-133b mimics (*P* < 0.01) and an miR-133b inhibitor (*P* < 0.01), respectively, into T24 cells (Figure 2).

### Bcl-w and Akt1 are putative targets of miR-133b according to bioinformatics results

To gain a greater understanding of the role of miR-133b in the pathogenesis of human bladder cancer, we looked for its potential downstream targets. Six bioinformatics algorithms were used to scan for possible targets and two identified: Bcl-w and Akt1. Bcl-w was predicted to be a target of miR-133b by five miRNA target prediction algorithms (Diana-microT, miRanda, miRWalk, PICTAR, TargetScan), and Akt1 was predicted to be a target of miR-133b by PITA.

### Luciferase reporter assay results

**Figure 3:**
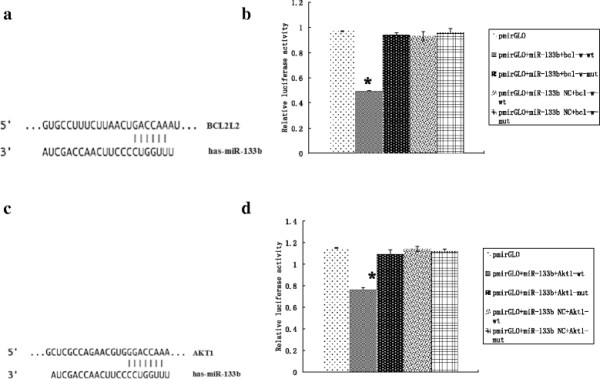
**Bcl-w and Akt1 as target genes of miR133b. (a)** Schematic diagram of the predicted miR-133b binding site in the Bcl-w 3′-UTR. **(b)** Luciferase reporter assays in untreated HEK-293 T cells, or cotransfected with wild type (wt) or mutant (mut) Bcl-w 3′-UTR, and miR-133b or miR-133b negative control (NC). *P < 0.01 **(c)** Schematic diagram of the predicted miR-133b binding site in the Akt1 3′-UTR. **(d)** Luciferase reporter assays in untreated HEK-293 T cells, or cotransfected with wild type (wt) or mutant (mut) Akt1 3′-UTR, and miR-133b or miR-133b negative control (NC). *P < 0.01.

To verify the binding of miR-133b to the 3′-UTR of Bcl-w and Akt1, a luciferase reporter assay was used. Results showed that miR-133b significantly decreased the luciferase activity of the Bcl-w (Figures 3a and b) and Akt1 (Figures 3c and d) 3′-UTRs in HEK-293 T cells (*P* < 0.01 for both), but not mutant sequences of the 3′-UTR of Bcl-w and Akt1. These results indicated that miR-133b bound specifically to the 3′-UTR of Bcl-w and Akt1 as predicted.

### Upregulated Bcl-w and Akt1 protein expression in bladder cancer tissue

**Figure 4:**
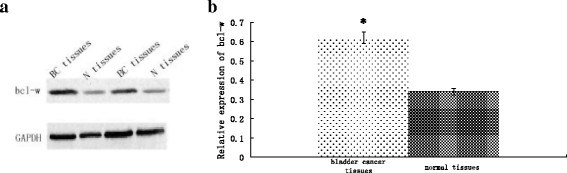
**Bcl-w expression is upregulated in bladder cancer tissue. (a)** Bcl-w protein expression was determined by Western blot in bladder cancer (BC) and normal (N) tissues. **(b)** Optical density values for combined Bcl-w protein relative to GAPDH in bladder cancer (BC) and normal (N) tissues. n = 41. *P < 0.01.

**Figure 5:**
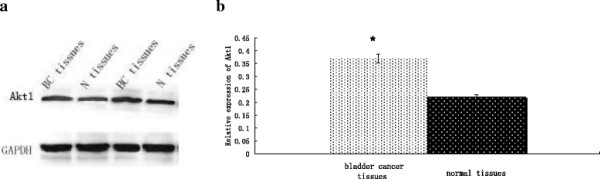
**Akt1 protein expression is upregulated in bladder cancer tissue. (a)** Akt1 protein expression was determined by Western blot in bladder cancer (BC) and normal (N) tissues. **(b)** Optical density values for combined Akt1 protein relative to GAPDH in bladder cancer (BC) and normal (N) tissues. n = 41. *P < 0.01.

Bcl-w and Akt1 protein expression was detected by Western blot in 41 bladder cancer specimens and adjacent normal tissues. We found that the combined expressions of Bcl-w (Figure 4) or Akt1 (Figure 5) proteins were significantly increased in bladder cancer specimens compared to adjacent normal tissues (*P* < 0.01 for both). These results suggest roles for Bcl-w and Akt1 in the pathogenesis of human bladder cancer.

### Effect of miR-133b on Bcl-w and Akt1 mRNA and protein expression in T24 human bladder cancer cells

**Figure 6:**
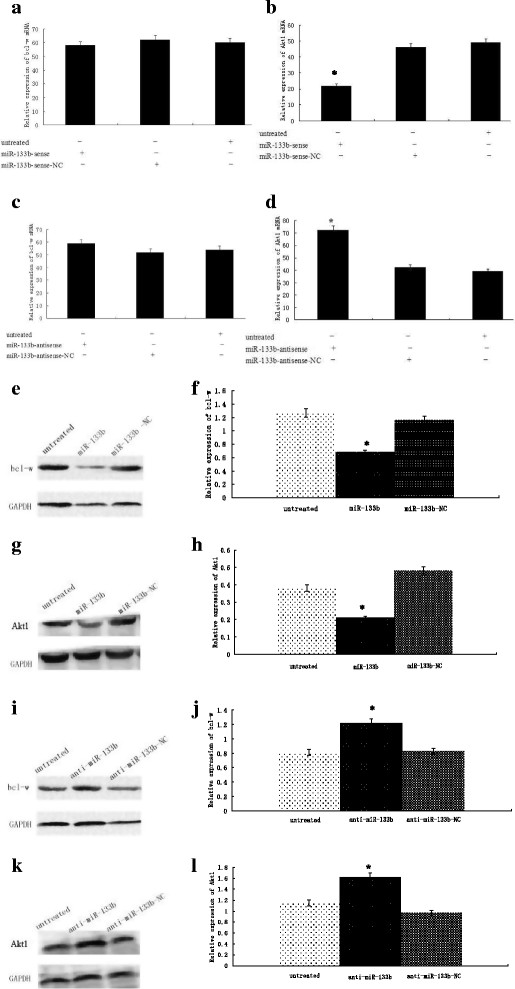
**Effect of miR-133b mimics and inhibitor on Bcl-w and Akt1 mRNA and protein levels in a T24 human bladder carcinoma cell line.** T24 cells were untreated or treated with miR-133b sense or miR-133b sense normal control (NC) **(a, b, e, f, g, h)** or with miR-133b antisense or miR-133b antisense normal control (NC) **(c, d, i, j, k, l)**. Bcl-w **(a, c)** Akt1 **(b, d)** mRNAs were measured by qRT-PCR. Bcl-w **(e, f, i, j)** or Akt1 **(g, h, k, l)** proteins were measured by Western blot. *P < 0.05.

The effects of miR-133b mimics or an inhibitor on levels of Bcl-w and Akt1 mRNA and protein in a human bladder cancer cell line, T24, were studied. Transfection of miR-133b-sense or miR-133b-antisense into T24 cells caused Akt1 mRNA expression to be significantly down-regulated or up-regulated (*P* < 0.05 for both), respectively (Figures 6b and d); however, there was no significant difference in Bcl-w mRNA expression (Figures 6a and c). Transfection of miR-133-sense into T24 cells caused Bcl-w (Figures 6e and ) and Akt1 (Figures 6g and h) protein expression to be significantly down-regulated (*P* < 0.05 for both; Figures 6e and f). Transfection of miR-133b-antisense into T24 cells caused Bcl-w (Figures 6i and j) and Akt1 protein (Figures 6k and l) expression to be significantly up-regulated (*P* < 0.05 for both). These results indicate that miR-133b down-regulates Akt1 mRNA and protein, and Bcl-w protein, but not its mRNA.

### MiR-133b inhibits cell proliferation of T24 cells

**Figure 7:**
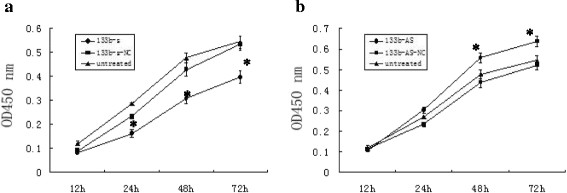
**Effect of miR-133b on proliferation of T24 cells. (a)** T24 cells were untreated or transfected with miR-133b-sense- negative control (NC) or miR-133b sense (S) for up to 72 h. **(b)** T24 cells were untreated or transfected with miR-133b-antisense-negative control (NC) or miR-133b antisense (AS) for up to 72 h. Cell proliferation was measured by CCK-8. *P < 0.05.

The effect of miR-133b on T24 cell proliferation was studied. Cell proliferation assays were performed at 12 h, 24 h, 48 h and 72 h after miR-133b-sense or miR-133b-antisense transfection of T24 cells. The cell proliferation of untreated or miR-133b-sense-NC transfected T24 cells was significantly greater compared to miR-133b-sense transfected cells (*P* < 0.05; Figure 7A). The cell proliferation of untreated or miR-133b- antisense -NC transfected T24 cells was significantly less compared to miR-133b-antisense transfected cells (*P* <0.05; Figure 7B). These results indicate that miR-133b expression in T24 cells inhibits their proliferation.

### MiR-133b promotes apoptosis of T24 cells

**Figure 8:**
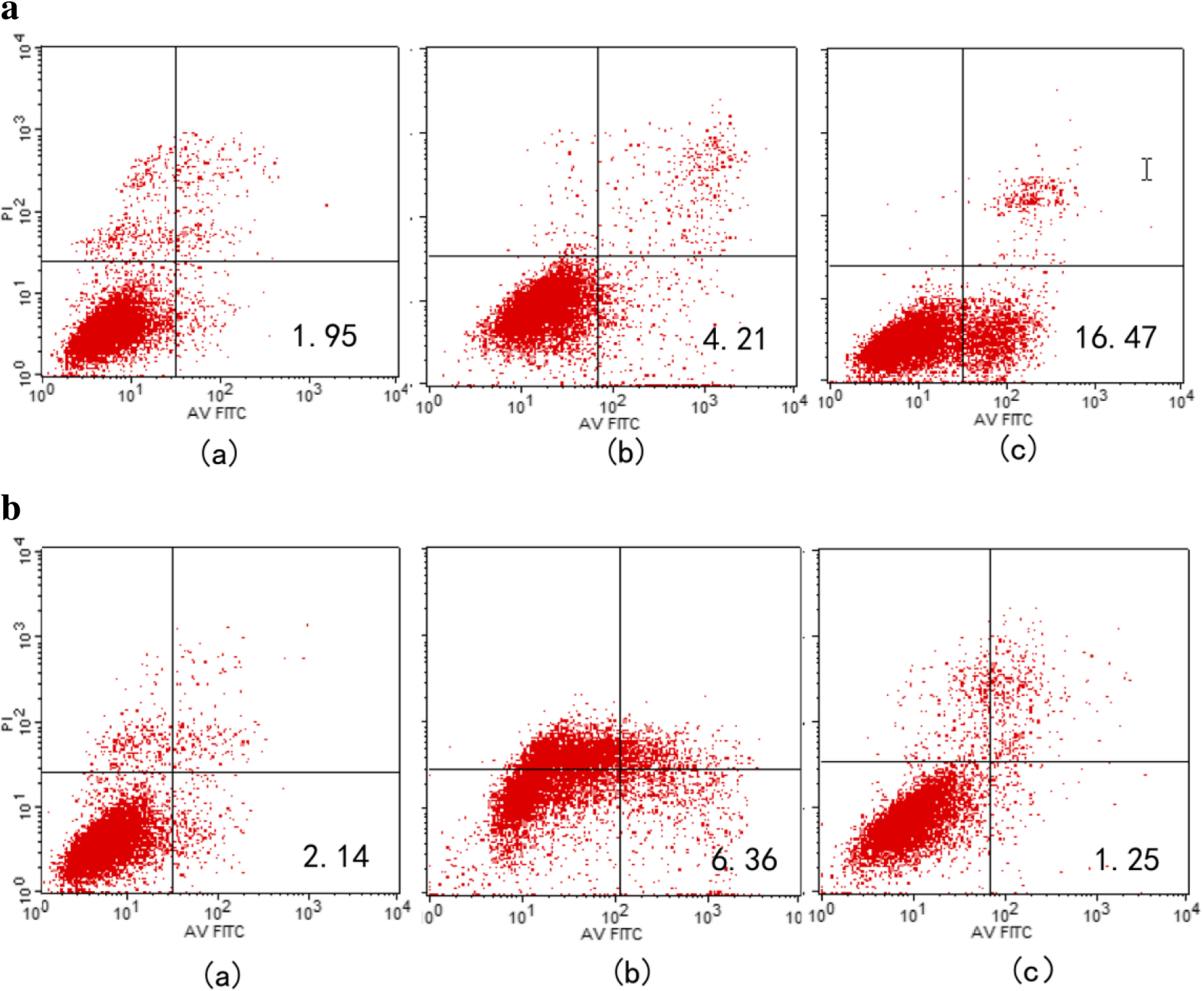
**The effect of miR-133b on T24 cell apoptosis. (a)** The effects of miR-133b-sense transfection on apoptosis of T24 cells: (a) untreated group, (b) miR-133b-sense-negative control (NC) group and (c) miR-133b-sense group. **(b)** The effects of miR-133b-antisense transfections on apoptosis of T24 cells: (a) untreated group, (b) miR-133b-antisense-normal control (NC) group and (c) miR-133b-antisense group. Apoptosis was measured by flow cytometric analysis of T24 cells stained with Annexin V-FITC 24 h after transfection.

In this study, Annexin V-FITC double staining flow cytometric analysis was performed to evaluate the effect of miR-133b on apoptosis in T24 cells. Apoptosis was determined 24 h after transfection. Compared with miR-133b-sense transfected cells (16.47%), the apoptosis rate of untreated cells (1.95%) was significantly less (*P* < 0.01; Figure 8a). Compared with miR-133b-antisense transfected cells (1.25%), the apoptosis rate of untreated cells (2.14%) was significantly greater (*P* < 0.05; Figure 8b). This data indicates that miR-133b promotes apoptosis in T24 cells.

## Discussion

The development of epigenetics, accounting for 99% of the human genome’s non-coding sequences, has attracted considerable attention by scientists, with miRNA research becoming a significant hotspot in recent years [20]-[22]. As non-coding, small molecule RNAs, miRNAs are involved in many important physiological and pathological processes, such as cell proliferation, development, differentiation, virus infection and tumorigenesis, and are widely dysregulated in various cancers [23]-[26]. MiRNA exerts its functions in a manner similar to oncogenes or tumor suppressor genes: tumor development and progression occur by the regulation of target genes. MiRNA-induced oncogene functions are often highly expressed in tumors, and miRNA-induced tumor suppressor genes often show low or no expression [27]-[29]. An miRNA can regulate multiple target genes, and a target gene can also be regulated by a number of miRNAs; both miRNA and target gene form a complex regulatory network, which plays an important role in the occurrence and development of human tumors.

Recent studies have shown that the expression of miR-133b is abnormal in many tumors. Masayuki et al. [30] found that the expression of miR-133b was significantly lower in esophageal squamous cell carcinoma; the proliferation and invasion of a carcinoma cell line could be inhibited after miR-133b transfection. In addition, they also found that miR-133b could affect the biological behavior of the tumor through regulating gene expression of FSCN1. Chiyomaru et al. [31] reported that miR-145 and miR-133a could influence the biological behavior of bladder cancer cells by also regulating the expression of FSCN1. We therefore speculated that miR-133b, whose nucleotide sequences are similar to those of miR-133a, may also play an important regulatory role in bladder cancer. Hu et al. [32] found that the expression of miR-133b was significantly decreased in colorectal cancer and in colorectal cancer cell lines SW-620 and HT-29; furthermore, it was found that miR-133b played an important role in vivo and in vitro by regulating the tyrosine kinase receptor, c-Met.

In this study, we examined expression levels of miR-133b in bladder cancer tissues from 41 patients by RT-PCR and found miR-133b was significantly down-regulated in bladder cancer tissues, which was consistent with previous research. Our results suggested miR-133b might regulate certain oncogenes to inhibit tumorigenesis. We further analyzed the relationship between the expression of miR-133b and clinicopathological factors, and found that a lower expression level of miR-133b was strongly associated with high-grade bladder cancer. However, miR-133b and gender, clinical stage and neoplasm recurrence showed no significant correlation. These findings support the idea that miR-133b may be an important regulator in bladder cancer.

In our study, CCK-8 tests and flow cytometry were used to evaluate the effects of miR-133b on cell proliferation and apoptosis of T24 cells, respectively. We found that miR-133b overexpression caused T24 cell growth inhibition and apoptotic enhancement. Thus, we hypothesized that miR-133b may function as a tumor suppressor, and the abnormal expression of miR-133b might be an important factor in bladder cancer incidence.

As a member of the BCL2 family, Bcl-w is able to inhibit cell apoptosis. Recently, Bcl-w was found to be highly expressed in some epithelial tumors. Lee et al. reported that Bcl-w could inhibit tumor cell apoptosis by blocking the SAPK/JNK pathway in gastric adenocarcinoma [33]. Bae et al. found that Bcl-w promotes gastric cancer cell invasion by inducing matrix metalloproteinase-2 expression via phosphoinositide 3-kinase (PI3 kinase), Akt, and Sp1 [34]. Guo et al. [35] reported that Bcl-w was observed in most human colon adenocarcinoma compared to normal tissues, which suggested that Bcl-w was involved in the regulation of colorectal cancer. The PI3 kinase-Akt pathway is frequently activated in cancer. Recent reports have identified that abnormal methylation of the Akt1 gene may be an early event during urocystic tumorigenesis and this should be further evaluated as a tumorigenesis marker for early detection of bladder cancer [36]. Askham et al. [37] reported the occurrence of an Akt1 mutation in bladder tumors. The Akt1 G49A (E17K) mutation was found in 2/44 (4.8%) of bladder cancer cell lines and 5/184 (2.7%) of bladder tumors. Cell lines expressing mutant Akt1 show constitutive Akt1 activation under conditions of growth factor withdrawal. In our study, the expression of Bcl-w and Akt1 proteins in 41 bladder cancer specimens and adjacent normal tissues was detected by Western blot assay. We found that Bcl-w and Akt1 proteins were significantly overexpressed in bladder cancer tissues versus adjacent normal tissues. This result is in accordance with previous research.

Melissa et al. reported that MiR-133b down-regulated the expression of MCL-1 and Bcl-w in lung adenocarcinoma cells, and overexpression of miR-133b increased the sensitivity of lung adenocarcinoma cells to gemcitabine [38]. We found the expression of Akt1 protein and mRNA in T24 cells was down-regulated and up-regulated, respectively, after transfection of miR-133b mimics or inhibitor. The expression of Bcl-w protein in T24 cells was down-regulated and up-regulated after transfection of miR-133b mimics or inhibitor, respectively, but there was no significant difference in Bcl-w mRNA expression.

In conclusion, in this paper we show that miR-133b expression is downregulated in bladder cancer tissues and is linked to high-grade bladder cancer. We show that miR-133b inhibits cell proliferation and induces apoptosis in a human bladder cancer cell line, T24. We found Bcl-w and Akt1 to be putative targets of miR-133b and show increased expression of these proteins in bladder cancer tissues. We show mi-R133b regulated Bcl-w and Akt1 in cultured T24 cells and we therefore speculated that miR-133b affects the biological behavior of bladder cancer by regulating the expression of Bcl-w and Akt1. However, mi-R133b’s specific role in bladder cancer and the mechanism(s) involved is unclear and requires further study.

## Materials and methods

### Specimens

Forty-one specimens of urothelial bladder cancer and adjacent normal tissue (more than 5 cm away from the tumor) were obtained from the Department of Urology, Shengjing Hospital of China Medical University, between December 2008 and December 2010. This study was approved by our institution’s Research Ethics Committee; informed consent was obtained from each patient. The age of patients ranged from 43 to 87 years, mean 62.3 ± 6.7 years, and included 29 males and 12 females. The histological grade and clinical stage were determined according to World Health Organization WHO (2004) pathological grading standards and the International Union Against Cancer UICC (2002) TNM clinical staging system.

### Cell culture and transfection

The human bladder cancer cell line T24 was obtained from the typical cell culture collection Committee of the Chinese Academy of Sciences Library (Shanghai, China). The cells were cultured in RPMI-1640 medium (HyClone, Logan, UT, USA) supplemented with 10% heat-inactivated FBS (JRH Biosciences, Lenexa, KS, USA), 100 U/ml penicillin, and 100 mg/L streptomycin. Cultures were maintained in a humidified atmosphere of 5% CO_2_ at 37°C. The following oligonucleotides were purchased from GenePharma (Shanghai, China): double-stranded miR-133b sense (mimics) and miR-133b-sense-negative control (NC); miR-133b -antisense (inhibitor) and miR-133b-antisense-negative control (NC); and sequences are as follows: miR-133b, 5′- UUUGGUCCCCUUCAACCAGCUA-3′, 5′-GCUGGUUGAAGGGGACCAAAUU-3′ and its NC 5′-UUCUCCGAACGUGUCACGUTT-3′, 5′- ACGUGACACGUUCGGAGAATT-3′; miR-133b-antisense, 5′-UAGCUGGUUGAAGGGGACCAAA-3′ and its NC 5′-CAGUACUUUUGUGUAGUACAA-3′. The day before transfection, cells were seeded in antibiotic-free medium. MiR-133b-sense, miR-133b-sense-NC, miR-133b-antisense and miR-133b-antisense-NC transfections were carried out using Lipofectamine 2000 in accordance with the manufacturer’s guidelines (Invitrogen). Untreated cells were designated as the control group.

### miR-133b target gene prediction

Computer-based programs were used to predict potential miR-133 targets. Using “has-miR-133b” as a search term, we queried PicTar (http://pictar.mdc-berlin.de/), TargetScan (http://www.targetscan.org/), miRWalk (http://www.umm.uni-heidelberg.de/apps/zmf/mirwalk/) miRanda (http://www.microrna.org/microrna/home.do), DIANA-microT (http://diana.cslab.ece.ntua.gr/microT/) and PITA (http://genie.weizmann.ac.il/pubs/mir07/mir07_data.html).

### Luciferase reporter assay

The target sequences of Bcl-w and Akt1 wild type 3′UTR were cloned into a luciferase vector which contained the *Renilla* luciferase gene. In addition, mutant 3′UTR was also cloned. HEK-293Tcells were cotransfected with miR-NC or miR-133b mimics using Lipofectamine 2000 (Invitrogen). Cells were collected 48 h after transfection and analyzed using the Dual-Luciferase Reporter Assay System (Promega, Madison, WI), and luciferase activity values were normalized relative to that of the *Renilla* luciferase internal control.

### Quantitative Real Time (RT)-PCR (qRT-PCR)

MiRNA of 41 bladder cancer specimens paired to adjacent normal bladder tissues were extracted using the mirVana miRNA Isolation Kit (Ambion, United States) according to the manufacturer’s instructions. Expression levels of miR-133b were analyzed by using stem-loop RT-PCR. MiR-133b stem-loop primer and U6 primer were purchased from Beijing Microread Gene Technology (Beijing, China). PrimeScript®RT reagent Kit and SYBR®Premix Ex Taq™ were purchased from TaKaRa (Otsu, Japan). U6 RNA was used as an internal control to normalize the relative abundance of miR-133b. The expression levels of Bcl-w and Akt1 mRNA were measured in T24 cells transfected with miR-133b-sense, miR-133b-sense-NC, miR-133b-antisense and miR-133b-antisense-NC. RT-PCR was performed using the following primers: miR-133b, 5′-GCGCTTTGGTCCCCTTC −3′/5′-CAGTGCAGGGTCCGAGGT-3′; U6, 5′-CTCGCTTCGGCAGCACA-3′/5′-AACGCTTCACGAATTTGCGT-3′; Akt1, 5′- GGTGATCCTGGTGAAGGAGA-3′*/*5′- CTTAATGTGCCCGTCCTTGT-3*′*[39]; and Bcl-w, 5′CACCCAGGT CTCCGATGAAC3′/5′TTGTTGACACTCTCA GCACAC3′ [40]. PCR cycles were as follows: 37°C for 15 min, 85°C for 5 s for reverse transcription, followed by 40 cycles of 95°C for 10 s, 95°C for 5 s, 61°C for 20 s. Cycle threshold (Ct) values were collected at the end of each PCR. Each sample was tested in triplicate, and the relative quantification equation (RQ = 2^-ΔΔCT^) was used to calculate the relative expression.

### Western blot analysis

Western blot analysis of Bcl-w and Akt1 proteins was conducted in 41 bladder cancer specimens paired to adjacent normal tissues, and also in a T24 cell line. Tissue and cell protein concentrations were determined using the Enhanced BCA Protein Assay Kit (Beyotime, Shanghai, China); equal amounts of proteins (20–30 μg) were separated by 15% SDS-polyacrylamide gel (SDS-PAGE) and transferred to PVDF membranes. After incubation with specific primary antibodies overnight at 4°C, membranes were further incubated for 1 hour with horseradish peroxidase-conjugated secondary antibodies. Integrated density values were analyzed using Fluor Chen 2.0 software (Olympus, Yokohama, Japan) and normalized to those of glyceraldehyde-3-phosphate dehydrogenase (GAPDH).

### Cell counting kit-8

Cell proliferation analysis was performed using the Cell Counting Kit-8 (CCK-8) (Tongren, Shanghai, China). The optimum reaction time of CCK-8 was determined to be 2.5 h. When 80% confluent, T24 cells (100 μL/well) were seeded into 96-well plates, and were left untransfected or transfected with miR-133b-sense or miR-133b-antisense, and further incubated for 12, 24, 48, and 72 hours using three replicates. Approximately 10 μL of tetrazolium salt, WST-8, was added to each well for 1 hour. The optical density (OD), at 450 nm, of each well was determined by microplate reader.

### Flow cytometric analysis

Apoptosis analysis was performed using a BioVision Annexin V-FITC reagent kit (Sigma-Aldrich; St. Louis, MO, USA) and flow cytometry. T24 cells were seeded onto 6-well plates and transfected with miR-133b-sense or miR-133b-antisense at 80% confluency. After transfection, cells were trypsinised and washed twice with PBS (2000 rpm/min, 5 min). Cells (1–5 × 10^5^) were collected, then 500 μL binding buffer, 5 μL Annexin V-FITC and 5 μL propidium iodide were added. Cells were incubated for 5–15 min after mixing, and flow cytometry carried out within an hour.

### Statistical analysis

Statistical analysis of data was performed using SPSS19.0 software. Statistical evaluation was performed using one-way analysis of variance (ANOVA; *P* < 0.05) and Student’s t-test. All data from experiments were expressed as mean ± SD unless otherwise stated.

## Abbreviations

miRNA: microRNA

miR133b: microRNA -133b

UTRs: Untranslated regions

NC: Negative control

CCK-8: Cell counting kit-8

OD: Optical density

## Competing interests

The authors declare that they have no competing interests.

## Authors’ contributions

XC and BW were involved in the concept and in the design, analysis and interpretation of the data and drafting of the manuscript. XC performed all the experiments and acquired the data. KW and ZX both participated in Western blotting assays and RT-PCR. SL and QL performed miR-133b target gene predictions, cell culture, transfections and dual luciferase reporter assays. DF and XW conducted cell counts with CCK-8, flow cytometric analysis and statistical analysis. All authors read and approved the final manuscript.
